# Inconclusiveness of psychometric testing of medication adherence questionnaires

**DOI:** 10.1007/s00228-024-03684-8

**Published:** 2024-04-22

**Authors:** Isabelle Arnet, Christiane Eickhoff, Laura J Sahm, Sabine Caloz, Michael Mittag, Martin Schulz, Samuel S Allemann

**Affiliations:** 1 Pharmaceutical Care, Pharmaceutical Sciences, University of Basel, Basel, Switzerland; 2 Department of Medicine, ABDA–Federal Union of German Associations of Pharmacists, Berlin, Germany; 3 Pharmaceutical Care Research Group, School of Pharmacy, University College Cork, Cork, Ireland; 4 Fachhochschule Nordwestschweiz, Windisch, Switzerland; 5 Institute of Pharmacy, Freie Universität Berlin, Berlin, Germany

**Keywords:** Medication adherence, Questionnaires, Reliability, Validity, Psychometric testing, Self-report

## Abstract

**Purpose:**

To propose a paradigm change for the validation procedures of medication adherence questionnaires.

**Methods:**

A total of 121 validation procedures of unique questionnaires for medication adherence were analyzed.

**Results:**

“Construct validity” and “internal consistency” were most often assessed, and test results varied largely. A more in-depth analysis indicated that the assessment of medication non-adherence included distinct but related constructs, such as the extent to which doses are missed, and the attempt to identify different facets of medication-taking behavior. Consequently, each construct requires a different measurement approach with different psychometric tests for establishing its validity and reliability.

**Conclusion:**

Results show that assessing the validity and reliability of adherence questionnaires with standard procedures including statistical tests is inconclusive. Refinement of the constructs of non-adherence is needed in pharmacy and medical practice. We suggest a distinction between the (i) *extent* of missed doses over the past 2 weeks, (ii) *modifiable reasons* for non-adherence behavior, and (iii) *unmodifiable factors* of non-adherence. Validation procedures and corresponding statistical methods should be selected according to the specific single constructs.

## Introduction

According to its most recent definition, medication adherence is a process by which patients use their medicines according to the recommendations [1], that is, a behavior that corresponds to the agreed regimen. Medication non-adherence is a well-described and huge challenge in the prevention, management, and treatment of patients. It is associated with increased healthcare costs [2], morbidity [3], and mortality [4]. As an example, poor adherence to antibiotic therapy contributes to the risk of increasing antimicrobial resistance with all of its associated consequences [5]. Thus, healthcare providers need accurate estimates of individual patients’ medication-taking behavior to ameliorate this problem and develop tailored interventions aimed at improving medication adherence. Three different phases of adherence that have been defined are initiation, implementation, and persistence [1], each is linked to different behaviors and thus requires different interventions when insufficient. There is no “gold standard” for assessing adherence [6]. Nonetheless, scales and self-report questionnaires remain a widely used method since they are inexpensive, easy to administer in any setting, and deliver immediate results. A large number of questionnaires to assess adherence have been developed in the past decades [7]. For all these instruments, the quality should be assured as a precondition to gather reliable data that can be used as a robust base for findings and interpretations.

The psychometric quality of a scale is defined by its validity and reliability [8]. One prerequisite to validity is that concepts exist and are represented by factors, and that variation in these factors affects the resulting scores on the questionnaire [9]. Depending upon the purpose of a questionnaire and how the items were developed, different statistical methods are used to establish validity and reliability [10].

In addition to statistical considerations, analytical approaches can be misleading, not least because a correlation between two variables does not mean that one is a measure of the other [11]. Thus, the appropriate statistical methods remain unclear [12], and results (in this context psychometric property) may not always be robust. For some older scales for example [13, 14], shortcomings have been detected including overly simplistic items or a scoring procedure that has not been subjected to adequate testing [15] making the use of these scales less reliable. Consequently, various frameworks and procedures to validate questionnaires have emerged [16]. Templates [17] or best practice guidelines [18] have been proposed, and standards such as the consensus-based COSMIN guidelines (COnsensus-based Standards for the selection of health Measurement INstruments) [19] have been developed. To facilitate further standardization, a COSMIN glossary of terms with their respective measurement properties has been defined [20] (Table 1).

**Table 1:**
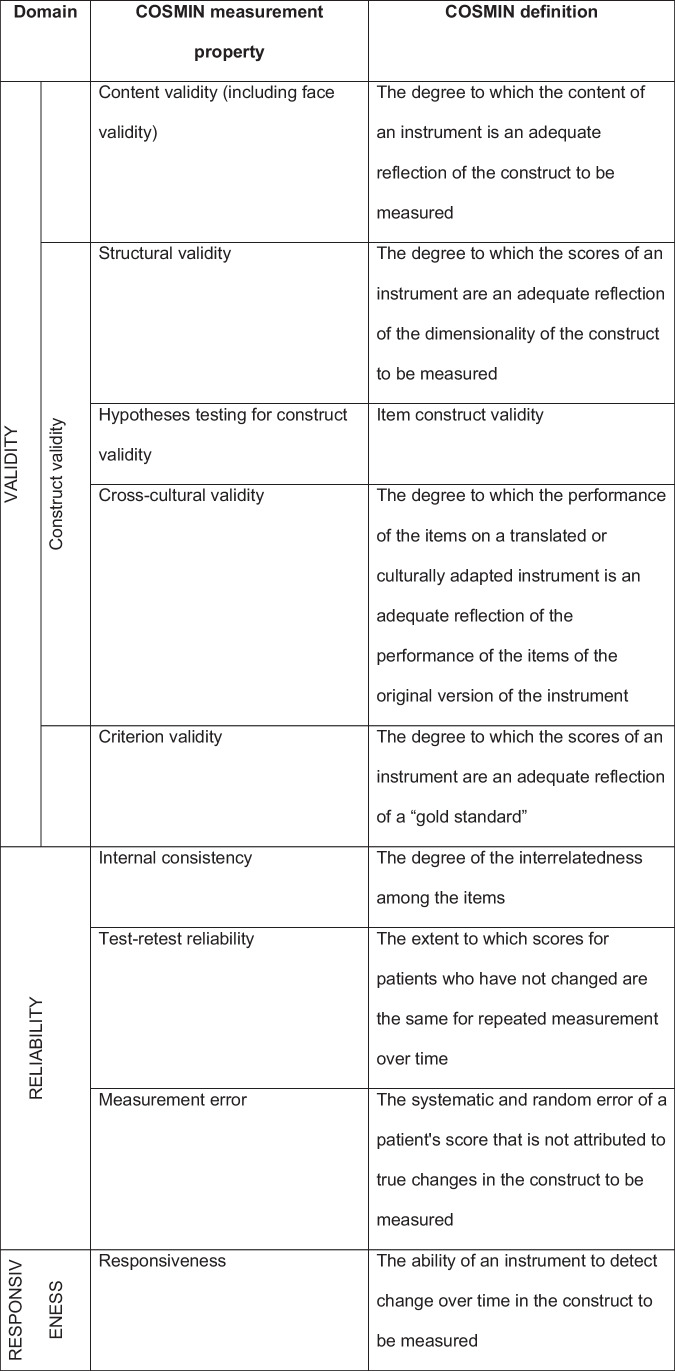
The nine COSMIN measurement properties with corresponding definitions. The domain “validity” contains three measurement properties: content validity, construct validity, and criterion validity. The domain “reliability” also contains three measurement properties: internal consistency, reliability, and measurement error. Adapted from [21] and [22]

However, despite these efforts, consensus among stakeholders is lacking in the selection of the optimal (self-report) instrument to assess medication adherence, although it is widely agreed that this measure should be included routinely in health records for primary care [23]. Unsurprisingly, many existing adherence questionnaires lack sufficient validity and reliability, as shown by Kwan [24]. This systematic review presents a summary of 121 validation procedures of unique questionnaires for medication adherence, published in 214 studies in 32 languages from 48 countries [24]. Based on the COSMIN guidelines, Kwan evaluated the methodological quality of each study and the psychometric results of each questionnaire to determine the level of evidence of the questionnaires and to recommend their use. However, Kwan did not summarize the measurement properties. Thus, our aims were (i) to calculate the frequency of the psychometric analysis of 121 validation procedures from Kwan’s review and (ii) to propose a paradigm change for the validation procedures of adherence questionnaires.

## Results

Out of the 121 validation procedures of Kwan’s review, we compiled a list of those measurement properties that were assessed (independent of quality) and how often the results were rated as sufficient (Table 2).

**Table 2:** Frequency of the psychometric analysis for validity, reliability, and responsiveness according to COSMIN used in 121 questionnaires on adherence. Adapted from [24]. Note that “criterion validity” was not assessed because there is no gold standard in the field of medication adherence. Also, note that “sufficient” was determined against COSMIN criteria for good measurement properties

	**Validity**				**Reliability**			**Responsiveness**
	Content validity	Hypotheses testing for construct validity	Structural validity	Cross-cultural validity	Test-retest reliability	Measurement errors	Internal consistency	
Frequency of psychometric analysis [%]	67.8%	88.4%	50.4%	1.6%	42.2%	0%	67.8%	38.8%
Frequency of psychometric analysis with sufficient results [%]	42.2%	46.3%	6.6%	0%	17.4%	0%	52.2%	18.2%

The measurement properties most often assessed were “construct validity” (that is, hypotheses testing for construct validity, structural validity, and cross-cultural validity) and “internal consistency” although “content validity” is considered to be the most important [25]. Cross-cultural validity was assessed for 1.6% of the questionnaires, although they were developed in 32 different languages. However, test results varied largely, and thus, a more in-depth interpretation is warranted.

If the assessment of non-adherence is reduced to the extent to which doses are missed, then the multitude of factors that underpin that behavior, as evidenced by 40 years of adherence research [26], are overlooked. Thus, in addition to an inquiry about the number of missed doses, questionnaires attempted to identify different facets of medication-taking behavior, and items elicited information regarding barriers to good adherence or beliefs about medicines [27]. Consequently, distinct but related constructs of non-adherence are measured, which have been summarized as the *extent* to which doses are missed and the *reasons* for missing doses [28]. Based on this dual conceptualization of non-adherence, each construct requires a different measurement approach with different statistical tests for its evaluation [29], i.e., for establishing its validity and reliability. Questionnaires that identify *reasons* for non-adherence mostly use validation methods that focus on content and construct validity [27]. However, because each reason for non-adherence is expected to stand alone and can only be correlated to others in case of redundancy [29], some authors propose to validate the *reasons* items individually and with fewer tests, e.g., reliability with test–retest and validity with cognitive interviews [28, 29].

## Discussion

In the current landscape, it is reasonable to claim that assessing the validity and reliability of medication adherence scales with standard procedures including statistical tests is inconclusive. Test results must be interpreted considering context and culture (among other variables) so that an oversimplified interpretation is avoided, particularly in the case of questionnaires that have not undergone rigorous methodological testing. Furthermore, suitable statistical methods must be chosen in line with the desired outcomes. Finally, we claim that a refinement of the constructs of non-adherence is needed in pharmacy and medical practice and research since questionnaire results should inform treatment decisions or enable tailored interventions to increase adherence.

Moreover, and following the tenets of Vrijens et al. [1], we suggest separating initiation and persistence from implementation. Not starting the treatment (non-initiation) or stopping the treatment too early (non-persistence) results in “missed doses,” but patients rarely think of them this way. When speaking with patients, the healthcare practitioners can ask whether the patient has discontinued, or never initiated, treatment. Thus, we suggest distinguishing three different constructs in the context of poor implementation. Firstly, the *extent* of missed doses over the past 2 weeks. In the literature, the time frame ranged from 1 day to 12 months [27]. Although a short recall period is said to minimize recall errors [30], the optimal time frame is unknown [31]. Nevertheless, forgetfulness is most likely to occur on a Saturday and/or Sunday, that is, when the daily rhythm is broken [32]. Thus, we believe that 2 weeks is an appropriate time frame being both short enough that patients will be able to recall and including a weekend. Secondly, *modifiable reasons* for non-adherence, that is, underlying barriers that may be possible to modify by the patient. They consist of unintentional (e.g., forgetfulness, inherent beliefs about treatment and medicines) or intentional reasons (e.g., lack of motivation, perceived need for medicine, or self-efficacy). Regarding adverse drug reactions, fear of side effects (that is, not actual side effects) is a well-known reason that leads to intentional non-adherence [33]. However, another reason is the presence of minimal but nonetheless uncomfortable side effects (for example feeling nauseous) usually requiring patients to seek advice from their doctor but may result in the patient skipping a dose or simply discontinuing their treatment [34]. Finally, drug shortages, particularly in low and middle-income countries, may put patients at risk of stopping their treatment for a while, independently of the healthcare system characteristics [35]. This may be inaccurately termed “voluntary” non-adherence. As cognitive and emotional factors guide people’s action [36], the emotion associated with the treatment, or the treatment-taking behavior, should be taken into consideration when creating items for medication adherence questionnaires (or instruments). To our knowledge, there is no core set of modifiable reasons (cognitions) for medication adherence. However, the theoretical domains framework (TDF) would appear to be both appropriate and comprehensive when describing the determinants of adherence behavior [37]. It captures 33 theories and 84 theoretical constructs related to behavior change [38] and matches behavior change techniques [39] to each of its 14 domains. Thirdly, there are *unmodifiable factors* (e.g., cognitive or sensory impairments, sociodemographic factors) negatively affecting adherence; these can be personal situational factors over which patients have little to no control. From the literature, it would appear that these unmodifiable factors are poorly correlated to non-adherence behaviors (e.g., suboptimal response to ACE-inhibitors by African Americans due to a low circulating plasma renin profile [40]); it would therefore be better to prioritize the development of interventions aimed at increasing adherence by means of changing modifiable factors. In addition, streamlining the items could be a practical advantage, that is, starting with the *extent* items could help identify patients with suboptimal levels of adherence. Following with the *modifiable reasons* items could enable to define targeted and feasible interventions.

As healthcare practitioners do not routinely have access to digital tools to be provided to patients, questionnaires are often filled out and scored by hand, on paper, so that every item adds to the burden of the healthcare practitioners, adding to the enormous time pressure. Thus, trade-off considerations are generally made by primary care physicians and pharmacists. However, if they choose to use a shorter questionnaire, they should keep in mind the three broad categories of abovementioned drivers. Then, if the initial questionnaire indicates that the patient is at behavioral risk, a more complete questionnaire might be useful to determine what the cause of non-adherence might be.

It is noteworthy to mention that adherence questionnaires in general can be broadly grouped into (i) those that purely center on inquiring into patient’s actual behavior (e.g., missed doses) and (ii) those that rather focus on patient’s attitudes (e.g., beliefs about medication). In this context, it is not surprising that none of the 121 questionnaires to measure non-adherence and analyzed by Kwan is satisfactory enough; otherwise, the best one would be the most used, especially after decades of research on non-adherence. The earlier questionnaires tended to consist only of items pertaining to the behavioral category, while more recent questionnaires, after Kwan’s analysis, have few or even no items that specifically ask about actual behavior. As an example, the SPUR (Social, Psychological, Usage, and Rational) questionnaire was developed in 2022 to profile type-2 diabetes patients and determine the risk of non-adherence via 27 items on attitudes [41]. However, the ideal adherence questionnaire should assess actual behavior while at the same time provide insights into its drivers, that is, the reasons behind non-adherence, as recently shown in the 15-STARS questionnaire [42].

## Conclusions

Assessing non-adherence with minimal efforts and without the need to delve into its reasons, i.e., with a simple questionnaire, may represent a significant step forward and might be acceptable for many healthcare practitioners. However, adherence questionnaires tackle different aspects of medication-taking behavior and therefore different constructs. Consequently, they are seldom comprehensive instruments. Thus, like there is no “one-size-fits-all” adherence questionnaire, there is no standard statistical test battery to determine the validity and reliability of these questionnaires. Validation procedures and corresponding statistical methods should match the single constructs. Thus, practitioners should carefully assess their need to identify the problem (i.e., non-initiation, non-implementation, or non-persistence) and only then determine its drivers with respect to the time and effort they are ready to expend. Finally, the interpretation of the test results deserves caution and pragmatism.

## Data Availability

No datasets were generated or analyzed during the current study.
